# Supplementary material for: Stomatal Ratio Showing No Response to Light Intensity in Oryza
Source: Plants (Basel). 2022 Dec 23;12(1):66. doi: 10.3390/plants12010066 (PMC9823486; doi:10.3390/plants12010066)
Supplement: Supplementary file 1 [file plants-12-00066-s001.zip › plants-2070244-supplementary.pdf]

Stomatal ratio showing no response to light intensity in Oryza

Tiange Wang[a], Linna Zheng[a], Dongliang Xiong[a], Fei Wang[a], Jianguo Man[a], Nanyan Deng[a], Kehui Cui[a], Jianliang Huang[a], Shaobing Peng[a] and Xiaoxia Ling[a]*

[a]*National Key Laboratory of Crop Genetic Improvement, Hubei Hongshan Laboratory, MOA Key Laboratory of Crop Ecophysiology and Farming System in the Middle Reaches of the Yangtze River, College of Plant Science and Technology, Huazhong Agricultural University, Wuhan, Hubei, 430070, China*

Corresponding author. Email: lingxiaoxia@mail.hzau.edu.cn

Supplementary materials

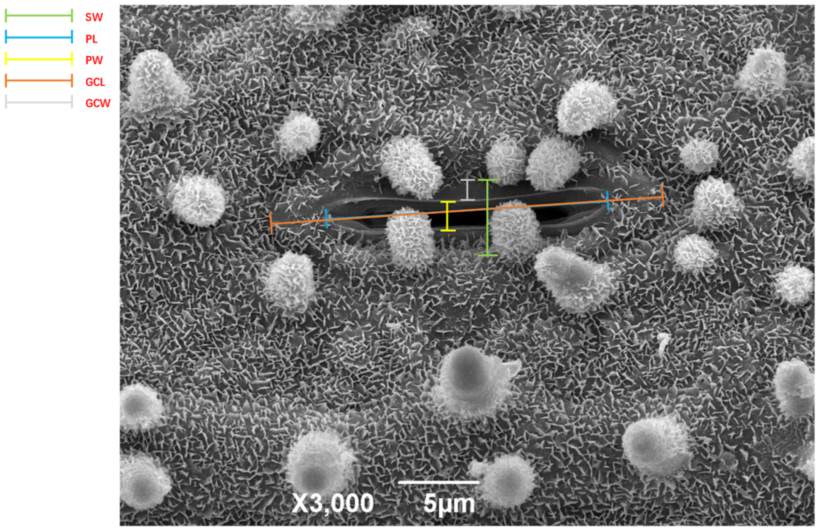

Figure S1 Diagram illustrating details of stomatal features.

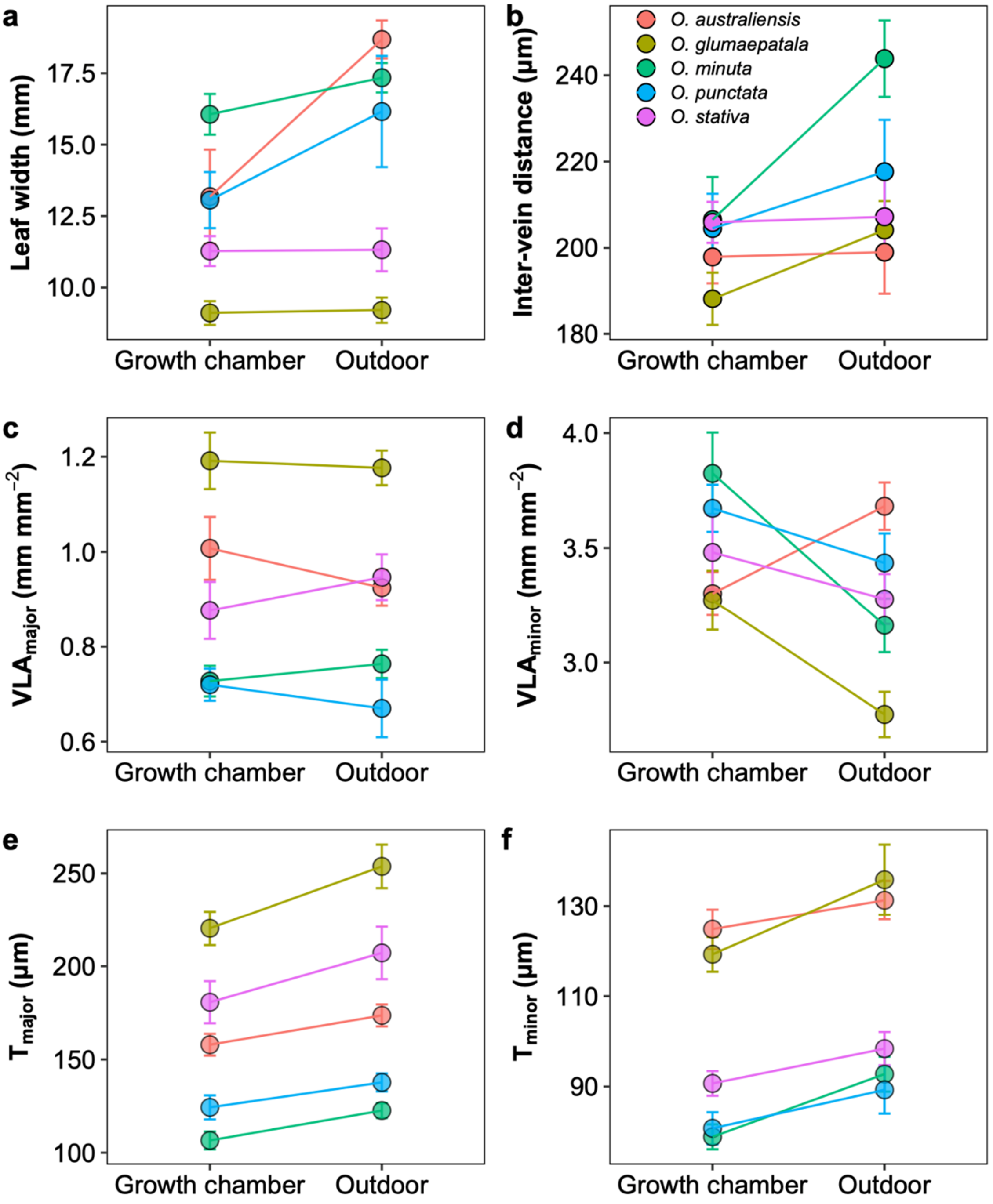

Figure S2. Plastic response of leaf anatomical traits to growth environments for five Oryza species. Mean ± SE values for leaf width (a), inter-vein distance (b), major vein length per area (VLAmajor; c), minor vein length per area (VLAminor; d), major vein thickness (Tmajor; e) and minor vein thickness (Tminor; f). All traits showed significant variation across species ($P < 0.01$, ANOVA, Table S1); N = 5 individual plants.

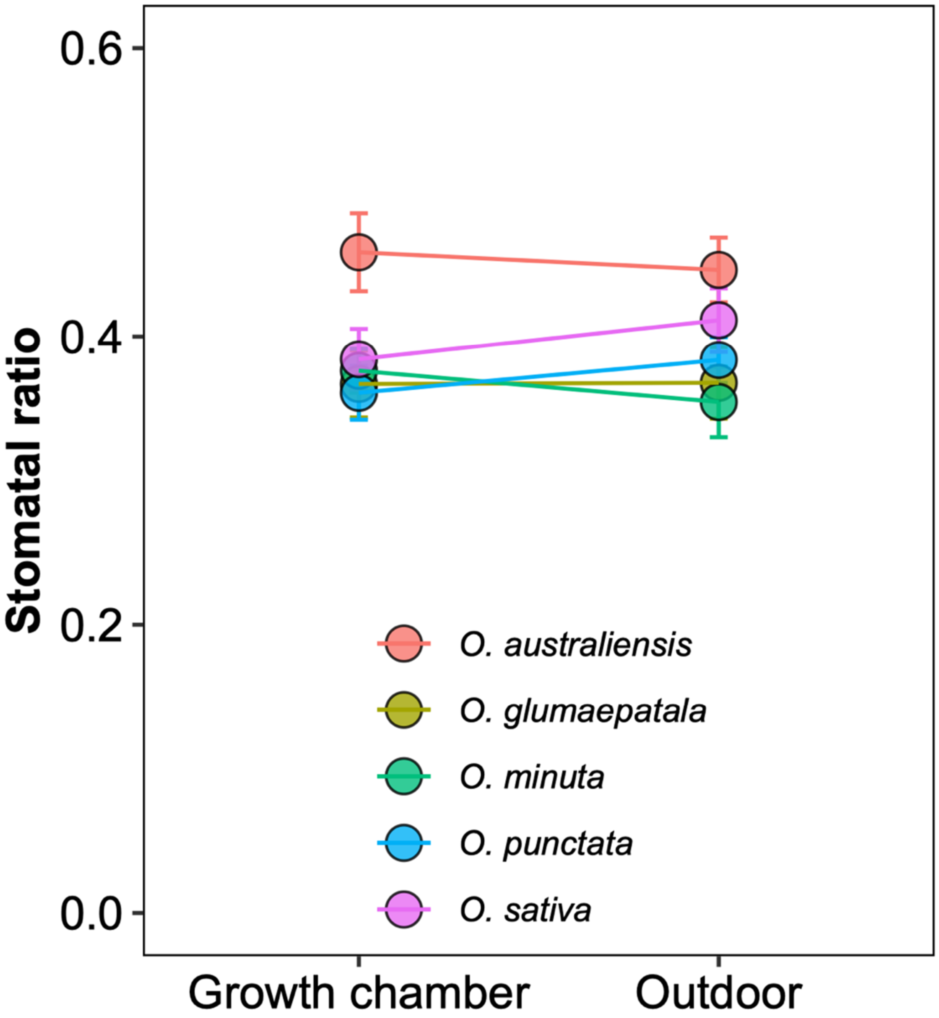

Figure S3. Stomatal ratio of five Oryza species grown in two conditions. Values were shown in Mean ± SE.

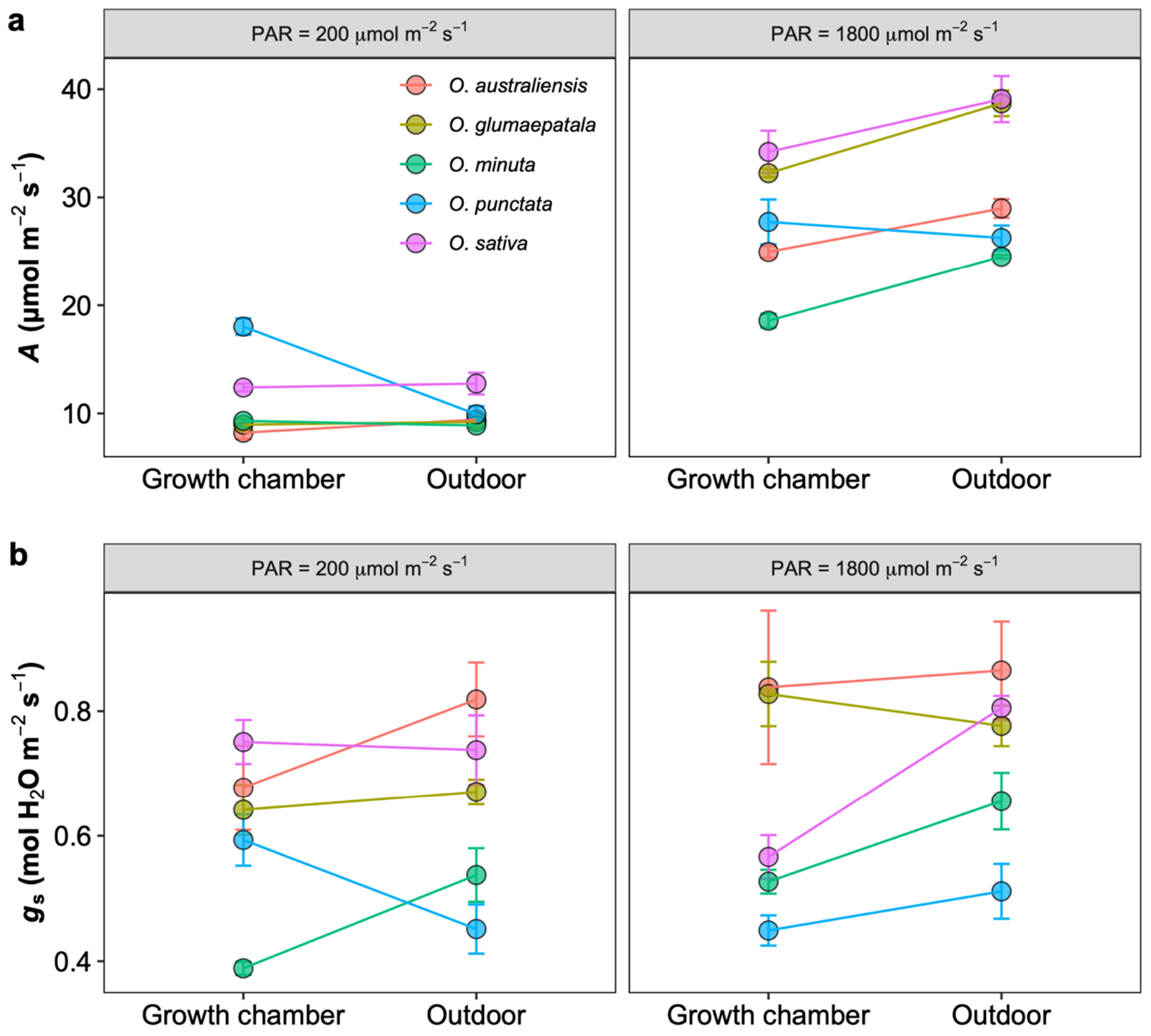

Figure S4. Plastic response of photosynthetic rate (A) and stomatal conductance (gs) to growth environments for five Oryza species.

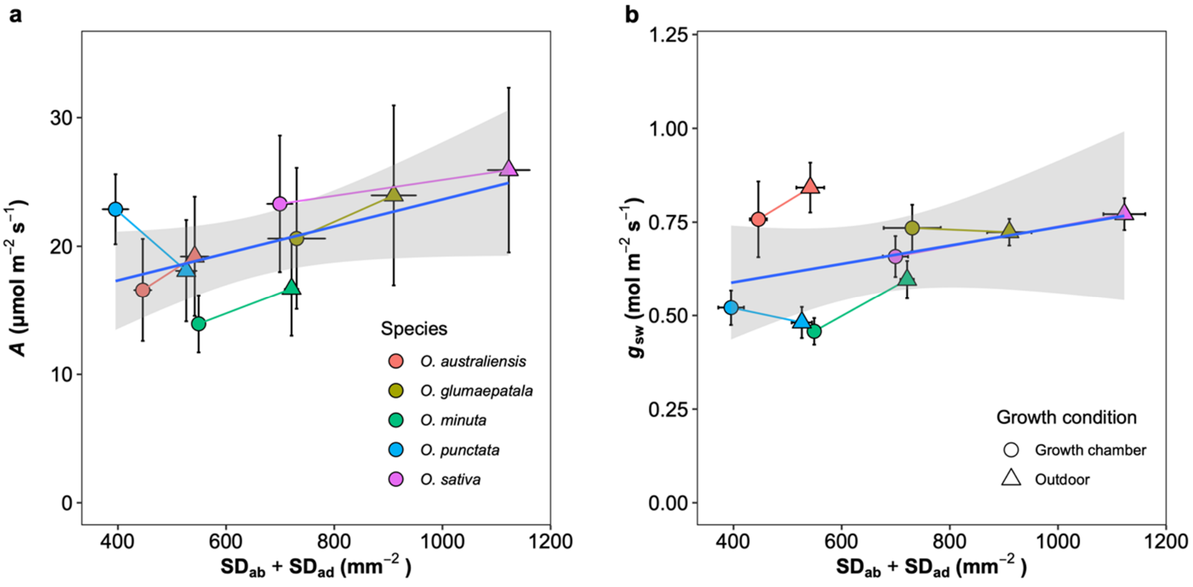

Figure S5. Correlation analysis between stomatal density and gas exchange parameters.

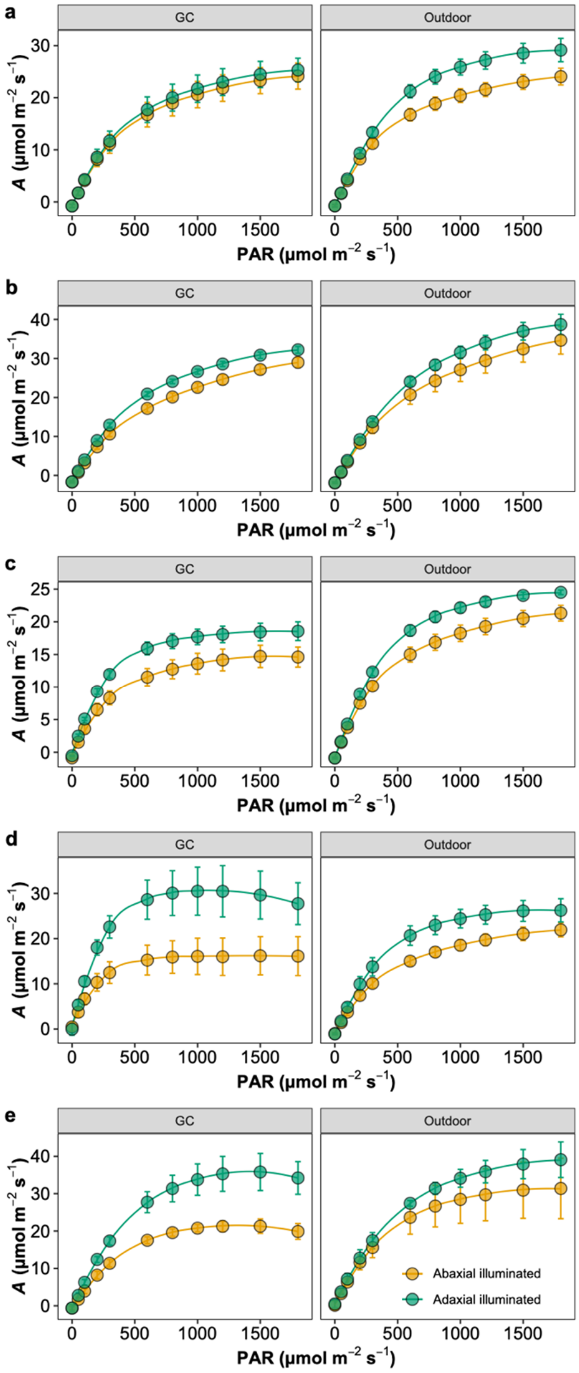

Figure S6 Light response curves of five Oryza species grown in two environments.
